# Neuroparacoccidioidomycosis: A 13-Year Cohort Study, Rio de Janeiro, Brazil

**DOI:** 10.3390/jof6040303

**Published:** 2020-11-20

**Authors:** Priscila Marques de Macedo, Eduardo Mastrangelo Marinho Falcão, Dayvison Francis Saraiva Freitas, Andréa d’Avila Freitas, Ziadir Francisco Coutinho, Mauro de Medeiros Muniz, Rosely Maria Zancopé-Oliveira, Rodrigo Almeida-Paes, Marcus Tulius Teixeira da Silva, Antonio Carlos Francesconi do Valle

**Affiliations:** 1 Clinical Research Laboratory on Infectious Dermatology, Evandro Chagas National Institute of Infectious Diseases, Fiocruz, Rio de Janeiro 21040-900, Brazil; eduardo.falcao@ini.fiocruz.br (E.M.M.F.); dayvison.freitas@ini.fiocruz.br (D.F.S.F.); antonio.valle@ini.fiocruz.br (A.C.F.d.V.); 2 Department of Inpatient Health Care, Evandro Chagas National Institute of Infectious Diseases, Fiocruz, Rio de Janeiro 21040-900, Brazil; andrea.freitas@ini.fiocruz.br; 3 Germano Sinval Faria School Health Center, Sergio Arouca National School of Public Health, Fiocruz, Rio de Janeiro 21040-900, Brazil; ziadir@centroin.com.br; 4 Mycology Laboratory, Evandro Chagas National Institute of Infectious Diseases, Fiocruz, Rio de Janeiro 21040-900, Brazil; mauro.muniz@ini.fiocruz.br (M.d.M.M.); rosely.zancope@ini.fiocruz.br (R.M.Z.-O.); rodrigo.paes@ini.fiocruz.br (R.A.-P.); 5 Clinical Research Laboratory on Neuroinfectious Diseases, Evandro Chagas National Institute of Infectious Diseases, Fiocruz, Rio de Janeiro 21040-900, Brazil; marcus.tulius@ini.fiocruz.br

**Keywords:** neuroparacoccidioidomycosis, paracoccidioidomycosis, neglected diseases, *Paracoccidioides*, central nervous system

## Abstract

Neuroparacoccidioidomycosis (NPCM) is a rare and severe clinical presentation of paracoccidioidomycosis (PCM). We performed a retrospective cohort study at the Evandro Chagas National Institute of Infectious Diseases (INI/Fiocruz), a reference center for PCM in the state of Rio de Janeiro, Brazil. All cases of PCM admitted to the INI/Fiocruz from January 2007 to December 2019 were reviewed. Eight (3.9%) among 207 patients met the diagnostic criteria for NPCM. The mean age was 44.6 years and the male:female ratio was 7:1. All cases presented multifocal disease, 5 (62.5%) the chronic form and 3 (37.5%) the acute/subacute form. All patients presented the pseudotumoral pattern and 6 (75.0%) had multiple lesions in the cerebral hemispheres. Seizures and motor symptoms were the most frequent clinical manifestations (50.0%, each). The treatment of choice was sulfamethoxazole/trimethoprim (SMZ-TMP) and fluconazole, in association (87.5%). Most patients responded well to the treatment. Sequela and death occurred in one (12.5%) patient, each.

## 1. Introduction

Neuroparacoccidioidomycosis (NPCM) is a rare and severe clinical presentation of paracoccidioidomycosis (PCM), a systemic mycosis caused by pathogenic dimorphic fungi belonging to the genus *Paracoccidioides*. Despite being one of the most important endemic mycoses in Latin America, PCM remains a neglected and not reportable disease [1]. Rare and atypical presentations of PCM are more susceptible to late diagnosis and, consequently, poor prognosis. PCM affecting the central nervous system (CNS) has substantial risks for sequelae [2].

This study aims to analyze epidemiological, clinical, laboratory, therapeutic, and prognostic features of NPCM cases from a 13-year cohort of a reference center for PCM in Rio de Janeiro, Brazil, an important endemic area of this mycosis, thus contributing to a better understanding of neurological aspects of PCM and to early diagnosis of NPCM.

## 2. Materials and Methods

### 2.1. Study Design and Ethical Statements

We performed a retrospective cohort study at the Evandro Chagas National Institute of Infectious Diseases (INI/Fiocruz), a reference center for PCM in the state of Rio de Janeiro, Brazil. Medical records were collected, and epidemiological, clinical, laboratory, therapeutic, and prognostic data were studied. The Research Ethics Committee of INI/Fiocruz has approved this study (Appreciation number 26066619.0.0000.5262). The patients’ data were anonymized/de-identified to protect patients’ privacy/confidentiality.

### 2.2. Patients

All cases of PCM admitted to the INI/Fiocruz from January 2007 to December 2019 were reviewed. The patients received a standard routine clinical evaluation, as previously described [3]. All NPCM cases identified in the study period were included.

### 2.3. Diagnostic Criteria

PCM diagnosis was performed through observation of *Paracoccidioides* spp. typical fungal structures on direct examination with 10% potassium hydroxide and/or histopathology, and/or fungal isolation in culture from clinical specimens of accessible lesions; and/or detection of specific serum antibodies against *Paracoccidioides* spp. using the Ouchterlony double immunodiffusion test (ID) [2]. Diagnostic criteria for NPCM were PCM diagnosis associated with neurological symptoms and neuroimaging lesions, which presented improvement after proper antifungal treatment. Lumbar puncture is not routinely performed in NPCM cases, except in cases of meningeal symptoms and for investigation of other differential diagnoses.

### 2.4. Treatment

Based on few published cases, the Brazilian Guidelines for the Clinical Management of PCM postulates sulfamethoxazole/trimethoprim (SMZ-TMP) as the treatment of choice for patients with NPCM [2]. The therapeutic regimen used routinely for NPCM cases in our center is SMZ-TMP, alone or associated to fluconazole (FCZ) [4], and/or amphotericin B (AMB) for severe lymphabdominal and/or disabsortive PCM cases.

### 2.5. Statistics

Descriptive statistics was used to depict the main features of patients’ data in this study. Frequency, means, and range of quantitative variables were calculated using the Microsoft Excel Professional Plus 2013.

## 3. Results

From January 2007 to December 2019, 207 PCM cases were diagnosed at INI/Fiocruz. Eight (3.9%) of them met the diagnostic criteria for NPCM. Table 1 depicts the epidemiological and clinical features of these cases. The mean age was 44.6 years (range: 22–56) and the male to female ratio was 7:1. Most patients (*n* = 6) received NPCM diagnosis at admission in our institution. At this moment, they were symptomatic from 3 to 12 months (mean: 7.6) and have sought unsuccessfully for diagnosis in other medical institutions. Cases 4 and 5 had low adherence to PCM treatment and developed NPCM during the follow-up at our institute. Patients did not present known immunodeficiencies.

Table 2 presents the laboratorial and radiological findings of the cases. All but one case presented confirmation of *Paracoccidioides* sp. presence by culture or visualization of the fungus in clinical specimens. Case 3 only presented a positive serology and, according to the Brazilian guidelines for PCM, this is a probable case of the mycosis [2]. This patient did not have any evidence of other infectious diseases (negative serological screening for histoplasmosis, cryptococcosis and search of alcohol acid resistant bacilli) and malignancies. All patients were screened for tuberculosis and other infectious diaseases included in the differential diagnosis of PCM and did not present any coinfection. Regarding the neurologic findings, all patients presented the pseudotumoral pattern and 6 (75.0%) had multiple lesions in the cerebral hemispheres. The parietal lobes were the most affected sites. The cerebellum was affected in one (12.5%) patient and the brain steam in two (25.0%). Seizures and motor symptoms were the most frequent clinical manifestations (50.0%, each). Three patients (cases 2, 4, and 8) were submitted to lumbar puncture due to the development of meningeal symptoms or for differential diagnosis, but their cerebrospinal fluid (CSF) did not present any alterations.

Table 3 summarizes relevant therapeutic and outcome data from these patients. In brief, sulfamethoxazole/trimethoprim (SMZ-TMP) was the treatment of choice in all cases, and fluconazole was used in association in most patients (87.5%). Amphotericin B was initially prescribed for cases 2 and 6 because they presented acute disseminated lymph abdominal forms at admission. For case 1, this choice was based on the high severity of this case. Satisfactory response to the treatment was observed in four patients, already discharged. Concerning these cases, all patients progressed with improvement of the neurological images and residual calcifications, except case 2, whose lesion completely disappeared. Three patients remain under antifungal treatment for PCM and still present active lesions. In case 8, there was an improvement of the lesions surrounding edema. One patient died after 9 months of hospitalization in our institution.

According to the recommendations of the Brazilian Guidelines for PCM [2], cases were followed at least for 24 months after treatment. Case 2 was recently consulted due to other symptoms not related to PCM and remained cured. The four patients who were considered cured had a mean treatment time of 41.3 (27–60) months.

## 4. Discussion

PCM is a systemic mycosis and the lungs are the usual portal of entry of *Paracoccidioides* into the human body. As for the other organs, the neurological involvement in PCM is secondary to fungal dissemination from a primary focus to the CNS through hematological and or lymphatic dissemination [5,6]. The exact mechanisms whereby *Paracoccidioides* spp. adhere and invade the blood-brain barrier are unknown. Particular virulence aspects of neurotropic fungal strains and specific genetic/immunological predispositions of the host are topics that deserve further studies.

This study reports eight NPCM cases among 207 patients from a 13-year PCM cohort at a reference center in Rio de Janeiro, Brazil. NPCM prevalence among PCM cases in the literature varies depending on the methodology used for investigation and the studied area. It ranges from 3.4% to 13.9% in some retrospective cohort studies [7,8], reaching 36.0% in necropsy evaluations [9,10]. Imaging studies using computerized tomography (CT) showed a prevalence of 12.5% for NPCM among PCM patients without neurologic symptoms [11]. The increase in the availability and quality of imaging tests, when combined with the epidemiology and clinical manifestations, enabled a greater number of diagnoses [12].

The CNS is most often affected in patients with the chronic (adult-type) form of the disease [13]. In this study, 5 (62.5%) patients presented the chronic form and 3 (37.5%) the acute/subacute (juvenile-type) form. A previous study also conducted by our group in the same endemic area, from 1991–2006, reported one (7.0%) patient with the acute/subacute form among 14 NPCM patients evaluated [4]. The higher frequency of neurological involvement in acute/subacute PCM cases found in this study can be related to changes recently observed in the clinical and epidemiological profile of PCM in the state of Rio de Janeiro [14]. Even in a rare presentation, the disease shows a marked professional bias, since 4 (50%) of the patients were rural workers.

**Figure 1:**
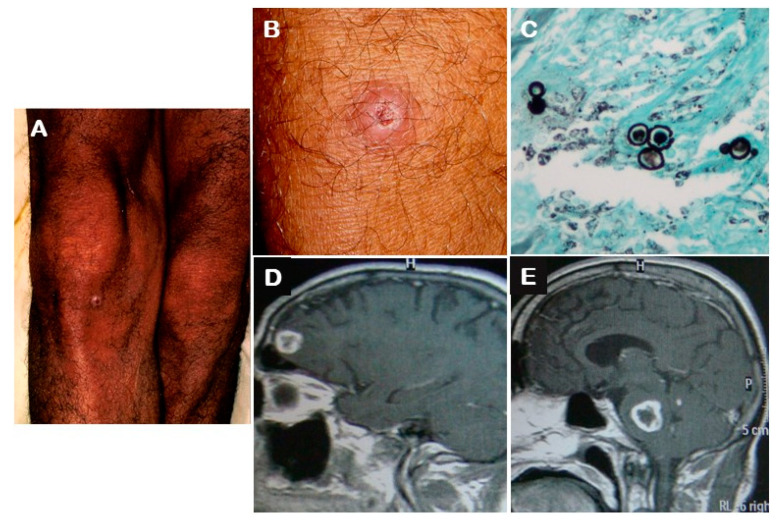
Neuroparacoccidioidomycosis in a patient with the chronic form of the mycosis: (**A**) A small round erythematous nodular skin lesion (around 1 cm) with central depression covered by crust on the right knee of the patient described as case 1; (**B**) Closer view of the lesion described in panel A; (**C**) Histopathological examination (Grocott silver stain) of a fragment of the skin lesion on the knee showing large budding yeast cells of *Paracoccidioides* sp.; (**D**) Magnetic resonance image showing a hyperintense (T1) round lesion with peripheral contrast enhancement and surrounding edema on the left frontal lobe (2 cm); (**E**) A similar brain lesion on the pons (2.5 cm).

NPCM is usually described as a multifocal disease, mainly associated with pulmonary or skin involvement [4,13,15]. In the present study, all patients presented multifocal disease and the PCM diagnosis was made for the most part through the mycological or histopathological examination of clinical samples from other accessible organs affected by the disease (Figure 1).

Magnetic resonance imaging and CT scan besides helping in the NPCM diagnosis are important to locate and characterize the neural lesions. The cerebral hemispheres are affected in 62.5–65.0%, the cerebellum in 25.0–33.3%, the brain steam in 8.3–25.0%, and the spinal cord in 4.0% [8,12]. Our findings are in accordance with the literature data showing predominance of the involvement of the cerebral hemispheres. The main radiological patterns are the pseudotumoral (87.5–90.0%), defined by the presence of parenchymal lesions with annular or nodular enhancement, and the meningeal (4.1–10.0%), characterized by inflammation of the leptomeninges or pachymeninges. The pseudotumoral lesions are frequently multiple (65.0%) [8,12]. All patients from this study presented pseudotumoral lesions, mostly multiple. Some hypotheses that could justify the most frequent occurrence of the pseudotumoral presentation when compared to the meningeal form are: (1) PCM is a granulomatous disease and the organic lesions are mostly characterized by a tumoral aspect being often misdiagnosed as neoplastic diseases; (2) PCM is an indolent disease that usually elicits poor inflammatory reaction thus explaining the scarce or misidentified cases of meningitis and encephalitis.

The most frequent clinical manifestations of NPCM observed in this study were seizures and motor symptoms, present in 4 (50.0%) patients each. One (12.5%) patient had headache and another patient had behavioral changes. On a previous study conducted by our group at the same reference center, the most common neurological manifestation was seizures (57.0%), followed by hemiparesis (29.0%), headache (21.0%), and ataxia (21.0%). Two (14.3%) patients presented also psychiatric symptoms [4]. Our results are in agreement with a systematic review that reported as main manifestations of NPCM: seizures, motor deficits, cerebellar signals, and headache [13].

The CNS involvement in PCM is poorly understood. Some conditions could be considered as risk factors, such as late diagnosis and low treatment adherence, allowing an increment in the capacity of fungal circulation and invasion. Furthermore, as previously mentioned, genetic predispositions of the host and some biological aspects from the fungal strain deserve further investigations. The role of an immunological deficit characterized by severe and disseminated disease in NPCM cases was suggested [16], but further studies did not show differences between NPCM and other PCM presentations regarding dissemination or a longer course of the disease [15].

Serological methods for antibody detection are important in the PCM diagnosis, especially in the context of inaccessible lesions, but mostly used for disease monitoring. Negative ID results at diagnosis are more frequent in the acute form (28.5 vs. 7.3%), probably due to the excess of antigens, immune complexes with occluded epitopes, asymmetric antibodies, or antibody levels below the sensitivity of the method [3,17]. In the present study, two cases (2 and 4) among three patients presenting the acute/subacute form had negative ID results whereas all patients with the chronic form presented positive ID results. It is noteworthy the limitation of serological methods to the diagnosis of PCM, due to *Paracoccidioides brasiliensis* and *Paracoccidioides lutzii* in different geographic regions [18]. Concerning the CNS involvement by these species, two previous studies conducted in Rio de Janeiro and Mato Grosso states, reported, respectively, two NPCM cases due to *P. brasiliensis* and no NPCM cases due to *P. lutzii*. Interestingly, all patients with PCM due to *P. lutzii* had the chronic type of the disease, mostly related to NPCM cases, whereas the chronic form occurred in 59% of the PCM cases due to *P. brasiliensis*. Both NPCM cases due to *P. brasiliensis* had the chronic form of the disease [19,20].

In this study, cases 4 and 5 were treated for PCM years before presenting NPCM, reinforcing that NPCM can occur in cases of PCM recurrence [4]. Case 4 did not complete the first treatment with amphotericin B and sulfamidics and retreated three times with ketoconazole, itraconazole, amphotericin B, and SMZ-TMP. Case 5 was treated for the first time with SMZ-TMP for 6 months and abandoned treatment.

Although the treatment of choice for severe PCM cases is amphotericin B, it does not have optimal penetration in the CNS, which makes SMZ-TMP the choice for NPCM treatment [2]. Our group observed a better prognosis in some patients who received SMZ-TMP and fluconazole, in association [4], thus we included this treatment schedule for NPCM cases at our center. Considering NPCM is rare, this level C recommendation is also based on the severity of this clinical presentation, the possible advantage of combining drugs as a therapeutic strategy in severe PCM cases, and the good penetration of fluconazole in the CNS. Furthermore, *Paracoccidioides brasiliensis* has shown to be highly sensitive to fluconazole in a previous study [21]. Before case 3 was admitted, he was misdiagnosed as neurotoxoplamosis and sulfadiazine was prescribed for 2 months. As he responded well, SMZ-TMP was prescribed alone. Voriconazole is a promising option to treat NPCM due to its effective in vivo action against *Paracoccidioides* spp. as demonstrated in an experimental rat model of the disease [22] and good penetration in the CNS, but its high cost is an obstacle [23,24]. Although itraconazole does not penetrate well in the CNS, some studies demonstrate its effectiveness, when prescribed in higher doses [4,25].

NPCM usually requires long time of treatment [4,8,13]. The patients who were considered cured in this study had a mean treatment time of 41.3 months. Case 5 presented the longest treatment period (96 months) due to persistent low adherence.

Even though NPCM has substantial risks for sequelae [2], only one patient from this study (case 6) progressed with this outcome, presenting permanent tetraparesis. The single fatal outcome occurred in case 1, probably due to multifactorial reasons, mainly late diagnosis along with multiple lesions including the greater lesion in the brain steam, which regulates vital cardiac and respiratory functions.

## 5. Conclusions

NPCM is a severe, poorly understood presentation of PCM and late diagnosis can lead to several complications, sequela, and death. The authors highlight the importance of studies characterizing NPCM cases contributing to the knowledge of this clinical presentation, helping to promote early diagnosis and better prognosis of the affected patients.

## Figures and Tables

**Table 1:** Epidemiological and clinical features of the neuroparacoccidioidomycosis (NPCM) cases from this study.

NPCM	Year *	Sex	Age	Origin	Risk Activity	NPCM Diagnosis	PCM Form	Neurologic Symptoms
Case 1	2008	M	51	RJ	Rural worker	At admission	Chronic	Seizures
Case 2	2009	F	30	RJ	None	At admission	Acute/subacute	Behavioral change
Case 3	2010	M	56	RJ	Rural worker	At admission	Chronic	Headache, diplopia, dizziness, vertigo
Case 4	2010	M	45	RJ	None	After 13 years follow-up	Acute/subacute	Seizures, ataxia, dysarthria, hypoacusia
Case 5	2012	M	49	RJ	None	After 14 years follow-up	Chronic	Seizures
Case 6	2014	M	22	RJ	None	At admission	Acute/subacute	Tetraparesis
Case 7	2017	M	55	BA	Rural worker	At admission	Chronic	Hemiparesis
Case 8	2019	M	49	ES	Rural worker	At admission	Chronic	Seizures, hemiparesis

Legend: NPCM (neuroparacoccidioidomycosis), PCM (paracoccidioidomycosis), M (male), F (female), RJ (Rio de Janeiro state), BA (Bahia state), ES (Espírito Santo state). * Year of NPCM diagnosis.

**Table 2:** Laboratorial and radiological findings of the NPCM cases from this study.

NPCM	Diagnostic Method	Radiologic Findings of Neurologic Lesions		PCM Serology	
		Type (Technique)	Local and Size	Admission	Discharge
Case 1	Direct examination (skin lesion)	Three hyperdense round lesions with surrounding edema (MRI)	Cortico-subcortical in the left frontal lobe (2 cm), parafalcine, right parietal lobe (1 cm), and the pons (2.5 cm)	Positive	-
Case 2	Culture (lymph node aspirate)	Single round mass effect lesion with contrast enhancement (CT)	Corpus callosum splenium (3 cm)	Negative	Negative
Case 3	Serology	Four nodular lesions with contrast enhancement and perilesional edema (MRI)	Cortico-subcortical in the frontal, parietal and occipital lobes, and cerebellum	1:8	Negative
Case 4	Culture (skin lesion)	Single round ring-enhancing lesion (CT)	Cortico-subcortical in the right frontal lobe, parafalcine (2 cm)	Negative	Negative
Case 5	Culture (sputum)	Four nodular lesions with ring-contrast enhancement (MRI)	Right inner capsule, left precuneus gyrus, right parietal operculum, left paracentral lobe (0.6 to 1.2 cm)	1:32	UT (1:4)
Case 6	Direct examination (lymph node aspirate)	Single hypodense lesion with contrast enhancement (MRI)	Mesencephalon (2.4 cm)	1:512	Negative
Case 7	Direct examination (skin lesion)	Four hypodense oval lesion with contrast enhancement (CT)	Cortico-subcortical (the greater 2.3 cm) in the right frontal lobe, parietal and temporal left lobe, and pons (1.3 cm)	1:16	UT (1:4)
Case 8	Histopathology (tongue)	Five round lesions with surrounding edema and contrast enhancement (CT)	Cortical-subcortical (the greater 3.1 cm) in the frontal and parietal left lobe, and right parietal lobe.	1:8	UT (1:1)

Legend: NPCM (neuroparacoccidioidomycosis), PCM (paracoccidioidomycosis), CT (computerized tomography), MRI (magnetic resonance imaging), UT (under treatment).

**Table 3:** Therapeutic and outcome data of the NPCM cases from this study.

NPCM	Drug	Time of Treatment (Months)	Time of Follow-Up (Months)	Outcome	Sequel
Case 1	AMB » SMZ-TMP + FCZ	12	-	Death	-
Case 2	AMB » SMZ-TMP + FCZ	27	96	Cure	None
Case 3	SMZ-TMP	30	31	Cure	None
Case 4	SMZ-TMP + FCZ	60	48	Cure	None
Case 5	SMZ-TMP + FCZ » FCZ ^1^	96	UT	UT	UT
Case 6	AMB » SMZ-TMP + FCZ	48	24	Cure	Tetraparesis ^2^
Case 7	SMZ-TMP + FCZ	35	UT	UT	UT
Case 8	SMZ-TMP + FCZ	14	UT	UT	UT

Legend: AMB (amphotericin B), SMZ-TMP (sulfamethoxazole/trimethoprim), FCZ (fluconazole), UT (under treatment). ^1^ Toxicity due to SMZ-TMP. ^2^ Need of walking support.
